# Association of Vitamin D Anabolism-Related Gene Polymorphisms and Susceptibility to Uterine Leiomyomas

**DOI:** 10.3389/fgene.2022.844684

**Published:** 2022-06-20

**Authors:** Shangdan Xie, Mengying Jiang, Hejing Liu, Fang Xue, Xin Chen, Xueqiong Zhu

**Affiliations:** Department of Obstetrics and Gynecology, The Second Affiliated Hospital of Wenzhou Medical University, Wenzhou, China

**Keywords:** vitamin D, uterine leiomyomas, single nucleotide polymorphisms, Dhcr7, NADSYN1

## Abstract

**Background:** Uterine leiomyomas (ULs) is the most common gynecological benign tumor in women. Our previous study showed that the phenomenon of vitamin D deficiency existed in patients with ULs. However, the association of vitamin D anabolism-related gene polymorphisms and susceptibility to ULs was unclear.

**Methods:** Vitamin D anabolism-related gene polymorphisms in 110 patients with ULs and 110 healthy controls were detected by sequencing and the differences of the 92 SNPs were analyzed in the two groups *via* chi-square test. To verify the association between the significantly different SNPs and the risk of ULs, the SNPs were genotyped in another 340 patients and 340 healthy controls. Additionally, an unconditional logistic regression model was conducted to calculate the odds ratio (OR) of ULs occurrence and the 95% confidence interval (CI), adjusting for age and BMI.

**Findings:** In sequencing samples, there were differences in DHCR7 rs1044482 C > T (*p* = 0.008) and NADSYN1 rs2276360 G > C (*p* = 0.025) between patients with ULs and healthy controls. DHCR7 rs1044482 was related to the susceptibility to ULs in validation samples (heterogeneous: adjusted OR = 1.967, *p* = 0.002; homogenous: adjusted OR = 2.494, *p* = 0.002; additive: adjusted OR = 1.485, *p* < 0.041; and dominant: adjusted OR = 2.084, *p* < 0.001). Stratified analysis further showed that the DHCR7 rs1044482 polymorphisms were associated with ULs risks in women over 40 and with 18.5–25.0 BMI. In contrast to the wild-type CG haplotype vectors, individuals with TC haplotypes had a higher risk of developing ULs.

**Interpretation:** The vitamin D anabolism-related gene DHCR7 rs1044482 C > T polymorphism was a risk factor of ULs, especially in patients over 40 with 18.5–25.0 BMI, while the relationship between NADSYN1 rs2276360 and ULs risk was not clear.

## Introduction

Uterine leiomyomas (ULs) are the most common gynecological benign tumor and characterized by the hyperplasia of uterine smooth muscle tissues (Stewart et al., 2017). Although ULs is not a lethal disease for most women cases, some patients will experience heavy menstrual abnormalities, abdominal lumps, increased leucorrhea and abdominal distension (Machado-Lopez et al., 2021). At present, the main treatment for symptomatic ULs is myomectomy or hysterectomy (Kotani et al., 2018). Despite such a high incidence of ULs, the etiology of ULs is not clear, which may explain the frequent recurrence of ULs after surgery. Thus, it is still necessary to further explore the pathogenesis of ULs for developing novel effective therapy.

Vitamin D (VitD) is a lipid-soluble steroid and is an important factor in regulating bone metabolism (Narvaez et al., 2020). In addition, VitD plays a central role in maintaining basic cell functions such as proliferation and differentiation (Samuel and Sitrin, 2008). Therefore, low levels of VitD represents a risk factor for several human diseases, including autoimmune, neurodegenerative, diabetes, and cancer (Marsh and Bulun, 2006; Goltzman et al., 2018; Bivona et al., 2019; Akutsu et al., 2020; Pittas et al., 2020; Ismailova and White, 2021). Of note, various studies have showed that vitamin D (VitD) deficiency may be closely bound up with occurrence of ULs (Singh et al., 2019; Srivastava et al., 2020). Our group has found that the levels of serum VitD3 in patients with ULs were significantly decreased compared with that in healthy controls (Li et al., 2020). Consistently, one study has found the expression level of VitD receptor in ULs was lower than that in nonneoplastic myometrial tissue (Lima et al., 2021). Several clinical trials have revealed that VitD intake in ULs cases with VitD deficiency prevented the growth of fibroids and related symptoms (Al-Hendy et al., 2015; Oskovi Kaplan et al., 2018). Contrarily, a recent randomized clinical trial reveals that VitD supplementation did not decrease the volume of ULs (Arjeh et al., 2020). Therefore, further elucidating the role of VitD deficiency in the development of ULs will provide evidence for the use of oral VitD in treating ULs.

VitD deficiency is a prevalence worldwide and can be caused by environmental factors such as diet, Sun exposure and stress (Dimakopoulos et al., 2019). Recently, accumulating evidence have reported a family cluster to VitD deficiency, which suggest the importance of genetic factors (Wang et al., 2010; Bahrami et al., 2018). This is mainly due to genetic variants involved in VitD metabolic pathways identified by whole-exome sequencing analysis (Alharazy et al., 2021). Therefore, it is proposed that mutation or loss of VitD metabolism-related genes may lead to VitD deficiency and subsequently promote the development of ULs. CYP27A1, GC, RXRA, CYP2R1, DHCR7, NADSYN1, VDR, CYP27B1, METTL1, ASIP, and CYP24A1 are important and functional genes in VitD anabolic pathway (Saponaro et al., 2020). A study shows that upregulation of VitD metabolic enzyme CYP24A1 level probably maintains the low VitD level in leiomyoma (Othman et al., 2018). However, there are few studies focused on the causes of decreased VitD expression in ULs patients (Saponaro et al., 2020). Therefore, the study aims to explore the relationship between single-nucleotide polymorphisms (SNPs) of these genes and susceptibility to ULs, which may provide a direction for exploring the causes of low VitD level in patients with ULs and the different effects of VitD anabolism-related gene polymorphisms on ULs risks by age and body mass index (BMI) stratification.

## Materials and Methods

### Subjects

The case-control study recruited 450 patients with ULs and 450 healthy married controls aged 27–58 years from The Second Affiliated Hospital of Wenzhou Medical University (WMU). The patients were confirmed to have ULs by pelvic ultrasound and the healthy controls experienced physical examination and showed no ULs by pelvic ultrasound. The exclusive criteria: 1) adnexal mass or endometrial polyps shown by pelvic ultrasound; 2) various severe diseases, including malignant tumors, cardiovascular diseases (myocardial infarction, cerebral infarction), endocrine diseases (abnormal parathyroid gland and type 2 diabetes), infectious diseases (tuberculosis), autoimmune disorders (Type 1 diabetes mellitus, systemic lupus erythematosus), hepatic or renal diseases; 3) other low vitD related diseases; 4) using vitD or calcium supplements within 6 months before study enrollment; 5) a past history of myomectomy or hysterectomy. This study was approved by ethics committee of The Second Affiliated Hospital of Wenzhou Medical University. Informed consent for involvement in the study was obtained from all participants. The following data were extracted for each case: age, BMI, white blood cells (WBC), red blood cells (RBC), alanine aminotransferase (ALT), aspartate aminotransferase (AST), total protein, carbamide, uric acid, triglyceride and total cholesterol. The BMI calculation formula was listed: weight (kg)/height (m)^2^. The stratification criteria for BMI referred to WHO criteria and was as follow: BMI <18.5 was underweight; 18.5 ≤ BMI <25.0 was normal weight; 25.0 ≤ BMI <30.0 was pre-obesity; 30.0 ≤ BMI <40.0 was obesity (Weir and Jan, 2021).

### DNA Isolation and Genotyping

TIANamp Blood DNA Kit [(TianGen Biotech, China) was utilized to collect genomic DNA from peripheral blood. The DNA purity and concentration was determined by The AUV absorption spectrophotometer (NanoDrop Technologies Inc., Thermo Fisher, United States)].

The DNA of 110 patients and 110 healthy controls was detected the bases at 92 sites of CYP27A1, GC, RXRA, CYP2R1, DHCR7, NADSYN1, VDR, CYP27B1, METTL1, ASIP, and CYP24A1 by NovaSeq6000 Sequencer (Illumina, United States). The probes for DHCR7 rs1044482 and NADSYN1 rs2276360 were purchased from Thermo Fisher (United States). The Genotyping qPCR PreMix was obtained from TianGen Biotech (China). The DNA of remaining 340 patients and remaining 340 healthy controls was applied in the genotyping assays for the two significant different SNPs between 110 patients and 110 controls. Genotyping analysis was conducted by the Taqman real-time polymerase chain reaction (PCR) method using a 7900 HT sequence detector system (Applied Biosystems, United States). The volume of each composition in total reaction system and amplification procedures required in PCR were listed in Supplementary Table S1. Besides, to evaluate the accuracy of genotyping outcomes, two positive controls and two negative controls were added in every 384-well plate.

### Statistical Analysis

The chi-square test was performed to calculate departure from Hardy-Weinberg equilibrium (HWE) for the all polymorphisms in 110 healthy controls. The measurement data with normal distribution were showed with mean ± standard deviation (SD) and compared by independent-samples t test, while those with abnormal distribution were described with quartile value and the differences were compared by nonparametric test. The relationship strength between the two SNPs (DHCR7 rs1044482 and NADSYN1 rs2276360) and ULs risk was evaluated via an unconditional logistic regression model, computed as crude and adjusted odds ratio (OR) and 95% confidence interval (CI). A *p* value < 0.05 indicated a statistical difference. Statistical analysis was conducted utilizing SPSS26.0 software.

## Results

### The Polymorphisms of Vitamin D Related Metabolic Genes in 110 Patients With Uterine Leiomyomas and 110 Healthy Controls

There was no difference in age between 110 patients with ULs (43.74 ± 5.15 years) and 110 healthy controls (43.17 ± 4.48 years, *p* = 0.334). The sequencing results of 59 loci of CYP27A1, GC, RXRA, CYP2R1, VDR, CYP27B1, METTL1, ASIP, and CYP24A1 in 220 subjects were shown in Supplementary Table S2 and there was no significant difference in the genotype distributions of 59 SNPs between patients and controls. The sequencing results of 33 SNPs of DHCR7 and NASDYN1 in 220 participants were shown in Table 1 and there were 11 loci with no mutations in healthy controls, so the relationship between the 11 SNPs and ULs occurrence was not analyzed. The genotype distributions of the remaining 22 SNPs in ULs-free controls followed HWE (*p* > 0.05). There was no significant difference in the genotype distribution of 20 loci between leiomyoma patients and controls, while there were differences in the genotype distributions of DHCR7 rs1044482 (*p* = 0.008) and NADSYN1 rs2276360 (*p* = 0.025) between the two groups. Therefore, this study enlarged the sample content to investigate the relationship between the polymorphisms of DHCR7 rs1044482 and NADSYN1 rs2276360 and the risk of ULs.

**TABLE 1:** The sequencing results of 33 SNPs of DHCR7 and NADSYN1 in 110 ULs and 110healthy controls.

Number	SNP	Position	Alleles	Gene	Case (N = 110)			Control (N = 110)			HWE	*P*
					0/0	0/1	1/1	0/0	0/1	1/1		
1	rs199506852	chr11: 71146468	G > A	DHCR7	109	1	0	109	1	0	0.962	1.000
2	rs781687341	chr11: 71146521	C > T	DHCR7	110	0	0	110	0	0	NA	NA
3	NA	chr11: 71146535	G > T	DHCR7	110	0	0	109	1	0	0.962	1.000
4	rs909217	chr11: 71146577	G > A	DHCR7	29	68	13	45	55	10	0.237	0.073
5	rs544442568	chr11: 71146681	G > A	DHCR7	110	0	0	109	1	0	0.962	1.000
6	rs760241	chr11: 71146691	A > G	DHCR7	1	41	68	6	37	67	0.690	0.151
7	NA	chr11: 71146810	C > G	DHCR7	109	1	0	110	0	0	NA	1.000
8	rs72954276	chr11: 71146837	C > T	DHCR7	110	0	0	109	1	0	0.962	1.000
9	rs75225632	chr11: 71146841	G > A	DHCR7	98	12	0	102	8	0	0.692	0.483
10	rs145901607	chr11: 71146862	G > A	DHCR7	106	4	0	100	9	1	0.146	0.212
11	rs1792268	chr11: 71146952	G > A	DHCR7	1	41	68	6	37	67	0.765	0.151
12	rs143811340	chr11: 71147010	G > A	DHCR7	109	1	0	110	0	0	NA	1.000
13	rs770925697	chr11: 71149986	G > A	DHCR7	110	0	0	110	0	0	NA	NA
14	rs949177	chr11: 71152461	A > G	DHCR7	0	30	80	4	34	72	0.969	0.107
15	NA	chr11: 71153351	C > T	DHCR7	109	1	0	110	0	0	NA	1.000
16	rs1790334	chr11: 71155153	A > G	DHCR7	0	30	80	4	34	72	0.996	0.097
17	rs1044482	chr11: 71155171	C > T	DHCR7	29	69	12	51	51	8	0.321	**0.008**
18	NA	chr11: 71155278	A > G	DHCR7	110	0	0	109	1	0	0.962	1.000
19	rs2276360	chr11: 71169547	G > C	NADSYN1	27	70	13	46	54	10	0.296	**0.025**
20	rs7950441	chr11: 71184678	A > C	NADSYN1	0	0	110	0	0	110	NA	NA
21	rs2276354	chr11: 71185479	T > C	NADSYN1	0	29	81	3	34	73	0.683	0.149
22	rs2186778	chr11: 71185518	T > C	NADSYN1	0	29	81	4	33	73	0.910	0.097
23	rs3819215	chr11: 71185582	G > A	NADSYN1	99	11	0	102	8	0	0.692	0.632
24	NA	chr11: 71188468	T > C	NADSYN1	110	0	0	109	1	0	0.962	1.000
25	rs2276353	chr11: 71189436	T > C	NADSYN1	0	29	81	3	34	73	0.683	0.149
26	rs149812928	chr11: 71189473	C > T	NADSYN1	110	0	0	109	1	0	0.962	1.000
27	rs138969547	chr11: 71191891	G > A	NADSYN1	110	0	0	108	2	0	0.923	0.498
28	rs147585323	chr11: 71193059	G > A	NADSYN1	109	1	0	110	0	0	NA	1.000
29	rs184748544	chr11: 71193951	C > A	NADSYN1	109	1	0	110	0	0	NA	1.000
30	rs765545198	chr11: 71194033	G > A	NADSYN1	109	1	0	110	0	0	NA	1.000
31	rs182956982	chr11: 71202892	G > A	NADSYN1	109	1	0	110	0	0	NA	1.000
32	rs371669981	chr11: 71209469	C > T	NADSYN1	109	1	0	110	0	0	NA	1.000
33	rs12282060	chr11: 71212387	G > A	NADSYN1	110	0	0	109	1	0	0.962	1.000

0/0 was homozygote without mutation; 0/1 was heterozygote with 1 mutation; 1/1 was homozygote with 2 mutations.

HWE, Hardy-Weinberg equilibrium; NA, not applicable.

### Association Between DHCR7 rs1044482 and NADYSYN1 rs2276360 and Uterine Leiomyomas Risk

340 patients and 340 healthy controls were recruited to verify the link strength between mutations of DHCR7 rs1044482 and NADSYN1 rs2276360 and occurrence of ULs. The demographic characteristics and some common indicators of ULs patients and controls were listed in Table 2. There was no difference in age, BMI, WBC, RBC, ALT, AST, total protein, carbamide, uric acid, triglyceride and total cholesterol between two groups (*p* > 0.05). As shown in Table 3, The values of HWE of DHCR7 rs1044482 and NADSYN1 rs2276360 demonstrated genetic balance in selected population. It was suggested that DHCR7 rs1044482 C > T was closely related to the occurrence of ULs (heterogeneous: adjusted OR = 1.967, 95% CI = 1.289–3.001, *p* = 0.002; homogenous: adjusted OR = 2.494, 95% CI = 1.389–4.477, *p* = 0.002; additive: adjusted OR = 1.485, 95% CI = 1.016–2.171, *p* < 0.041; and dominant: adjusted OR = 2.084, 95% CI = 1.396–3.110, *p* < 0.001). In addition, NADSYN1 rs2276360 G > C might be a risk factor of ULs (homogenous: adjusted OR = 2.020, 95% CI = 1.121–3.640, *p* = 0.019; additive: adjusted OR = 1.079, 95% CI = 0.726–1.606, *p* = 0.706; and recessive: adjusted OR = 1.727, 95% CI = 1.008–2.960, *p* = 0.047). When the two SNPs were analyzed in combination, the vectors of two risk genotypes could raise the susceptibility to ULs (adjusted OR: 1.856, 95% CI = 1.197–2.877, *p* = 0.030).

**TABLE 2:** The information of selected variables of validated ULs patients and controls.

Variables	Case (N = 340)			Control (N = 340)			*P*	
	Medium ±SD			Medium ±SD				
Age	39.90 ± 4.80			39.99 ± 4.38			0.770	
BMI	22.46 ± 2.93			21.93 ± 2.61			0.282	
**Variables**	**Medium**	**P25**	**P75**	**Medium**	**P25**	**P75**	***Z***	***P***
WBC	5.72	4.85	6.84	5.72	4.81	6.72	−0.005	0.996
RBC	4.37	4.15	4.61	4.40	4.19	4.62	−1.052	0.293
ALT	13.00	11.00	17.00	14.00	11.00	19.00	−1.645	0.100
AST	17.00	15.00	20.00	18.00	15.00	20.00	−0.697	0.486
Total protein	73.50	71.20	76.20	73.70	70.90	76.40	−0.667	0.505
Carbamide	4.40	3.80	5.20	4.50	3.90	5.10	−1.312	0.190
Uric acid	269.00	231.00	313.50	277.00	240.00	310.00	−1.039	0.299
Triglyceride	0.91	0.69	1.30	0.98	0.73	1.33	−0.963	0.335
Total cholesterol	4.63	4.06	5.07	4.62	4.19	5.09	−0.487	0.627

BMI, body mass index; WBC, white blood cell; RBC, red blood cell; ALT, alanine aminotransferase; AST, aspartate aminotransferase.

Normally distributed data is represented by Medium ± SD, non-normally distributed data is represented by quartile, and Z is the statistical value of nonparametric test.

**TABLE 3:** Association between the two SNPs and ULs by logistic regression analyses.

Allele	Case (N = 340)	Control (N = 340)	Crude OR (95% CI)	*p*	Adjusted OR (95% CI)^a^	*p* ^a^
DHCR7 rs1044482 C > T						
C	349 (51.32%)	402 (59.12%)	References		References	
T	293 (43.09%)	238 (35.00%)	1.418 (1.134–1.773)	**0.002**	1.651 (1.251–2.178)	**< 0.001**
NA	38 (5.59%)	40 (5.88%)		NA		NA
NADSYN1 rs2276360 G > C						
G	325 (47.79%)	382 (56.18%)	References		References	
C	253 (37.21%)	218 (32.06%)	1.364 (1.080–1.723)	**0.009**	1.430 (1.073–1.906)	**0.015**
NA	102 (15.00%)	80 (11.76%)		NA		NA
Genotype	Case (N = 340)	Control (N = 340)	Crude OR (95% CI)	*p*	Adjusted OR (95% CI)^a^	*p* ^a^
DHCR7 rs1044482 C > T, HWE = 0.106						
CC	92 (27.06%)	133 (39.12%)	References		References	
CT	165 (48.53%)	136 (40.00%)	1.754 (1.237–2.488)	**0.001**	1.967 (1.289–3.001)	**0.002**
TT	64 (18.82%)	51 (15.00%)	1.814 (1.152–2.856)	**0.010**	2.494 (1.389–4.477)	**0.002**
NA	19 (5.59%)	20 (5.88%)		NA		NA
Additive			1.431 (1.048–1.954)	**0.024**	1.485 (1.016–2.171)	**0.041**
Dominant	229 (67.35%)	187 (55.00%)	1.770 (1.275–2.459)	**0.001**	2.084 (1.396–3.110)	**< 0.001**
Recessive	257 (75.59%)	269 (78.12%)	1.313 (0.875–1.971)	0.188	1.689 (0.994–2.870)	0.053
NADSYN1 rs2276360 G > C, HWE = 0.178						
GG	95 (27.94%)	127 (37.35%)	References		References	
GC	135 (39.71%)	128 (37.65%)	1.410 (0.984–2.020)	0.061	1.336 (0.864–2.067)	0.193
CC	59 (17.35%)	45 (13.24%)	1.753 (1.095–2.805)	**0.019**	2.020 (1.121–3.640)	**0.019**
NA	51 (15.00%)	40 (11.76%)		NA		NA
Additive			1.178 (0.851–1.631)	0.324	1.079 (0.726–1.606)	0.706
Dominant	194 (57.06%)	173 (50.88%)	1.499 (1.072–2.097)	**0.018**	1.494 (0.993–2.247)	0.054
Recessive	230 (67.65%)	255 (75.00%)	1.454 (0.949–2.228)	0.086	1.727 (1.008–2.960)	**0.047**
Combined effect of risk genotypes^b^						
0	78 (22.94%)	111 (32.65%)	References		References	
1	19 (5.59%)	25 (7.35%)	1.082 (0.557–2.099)	0.817	1.198 (0.529–2.716)	0.665
2	181 (53.24%)	151 (44.41%)	1.706 (1.189–2.448)	**0.004**	1.856 (1.197–2.877)	**0.006**
NA	62 (18.24%)	53 (15.59%)		NA		NA

a Adjusted for age and BMI.

b Risk genotype was with DHCR7 rs1044482 CT/TT, and NADSYN1 rs2276360 GC/CC.

The results were in bold if *p* value <0.05.

OR, odd ratio; CI, confidence interval; HWE, Hardy-Weinberg equilibrium; NA, not applicable.

### Stratified Analysis and Haplotype Analysis

As shown in Table 4, the relationship of DHCR7 rs1044482 C > T and NADSYN1 rs2276360 G > C and ULs susceptibility was further studied via stratified analysis. The DHCR7 rs1044482 CT/TT genotypes (*p* = 0.001) and the NADSYN1 rs2276360 GC/CC genotypes (*p* = 0.017) could increase the risk of ULs among the women over 40 years old, respectively. Besides, among the participants with 18.5–25.0 BMI, the susceptibility to ULs was raised in the women with DHCR7 rs1044482 CT/TT genotypes (*p* = 0.001) or NADSYN1 rs2276360 GC/CC genotypes (*p* = 0.034). Furthermore, the haplotypes of the two SNPs were investigated in Table 5. In comparison of the reference haplotype CG, CC (adjusted OR = 1.640, 95% CI = 1.193–2.256, *p* = 0.002, TG (adjusted OR = 1.374, 95% CI = 1.009–1.872, *p* = 0.012) and TC (adjusted OR = 1.851, 95% CI = 1.441–2.379, *p* < 0.001) all increased ULs risk, respectively.

**TABLE 4:** Stratification analysis of risk alleles and genotypes with ULs susceptibility.

Characteristics	rs1044482 (Case/Control)		OR (95%CI)	*p*	rs2276360 (Case/Control)		OR (95%CI)	*p*
	C	T			G	C		
Age (year)								
<40	190/203	140/113	1.324 (0.964–1.818)	0.083	175/190	117/106	1.198 (0.859–1.673)	0.288
≥40	159/199	153/125	1.532 (1.118–2.099)	**0.008**	150/192	136/112	1.554 (1.119–2.160)	**0.009**
BMI								
<18.5	15/27	11/7	2.829 (0.906–8.832)	0.073	14/23	10/9	1.825 (0.596–5.590)	0.292
18.5 ≤ BMI <25.0	194/220	154/110	1.588 (1.163–2.168)	**0.004**	181/207	133/105	1.449 (1.047–2.004)	**0.025**
25.0 ≤ BMI <30.0	41/31	37/21	1.332 (0.655–2.710)	0.429	36/27	30/23	0.978 (0.468–2.045)	0.953
30.0 ≤ BMI <40.0	1/3	3/221	9.000 (0.367–220.927)	0.178	0/3	0/1	NA	NA
**Characteristics**	**rs1044482 (Case/Control)**		**OR (95% CI)**	***p***	**rs2276360 (Case/Control)**		**OR (95% CI)**	***p***
	**CC**	**CT + TT**			**GG**	**GC + CC**		
Age (year)								
<40	56/67	109/91	1.433 (0.913–2.250)	0.118	54/63	92/85	1.226 (0.684–2.196)	0.494
≥40	36/66	120/96	2.292 (1.408–3.729)	**0.001**	41/64	102/88	1.809 (1.114–2.938)	**0.017**
BMI								
<18.5	6/10	7/7	1.667 (0.388–7.153)	0.492	6/8	6/8	1.000 (0.224–4.468)	1.000
18.5 ≤ BMI <25.0	51/78	123/87	2.162 (1.383–3.382)	**0.001**	54/72	103/84	1.635 (1.037–2.578)	**0.034**
25.0 ≤ BMI <30.0	10/8	29/18	1.289 (0.429–3.872)	0.651	10/6	23/19	0.726 (0.223–2.365)	0.595
30.0 ≤ BMI <40.0	0/1	2/1	NA	1.000	0/1	0/1	NA	NA

The results were in bold if *p* value <0.05.

OR, odd ratio; CI, confidence interval.

**TABLE 5:** The frequency of inferred haplotypes of the two SNPs based on observed genotypes and their association with the risk of ULs.

rs1044482	rs2276360	Case (N = 340)	Control (N = 340)	Crude OR (95% CI)	*p*	Adjusted OR (95% CI)^a^	*p* ^a^
C	G	231 (41.25%)	301 (50.50%)	References		References	
C	C	80 (14.29%)	77 (12.92%)	1.456 (1.128–1.880)	**0.004**	1.640 (1.193–2.256)	**0.002**
T	G	82 (14.64%)	76 (12.75%)	1.401 (1.085–1.810)	**0.010**	1.374 (1.009–1.872)	**0.012**
T	C	167 (29.82%)	142 (23.83%)	1.587 (1.297–1.942)	**< 0.001**	1.851 (1.441–2.379)	**< 0.001**

a Adjusted for age and BMI.

The results were in bold if *p* value <0.05.

OR, odd ratio; CI, confidence interval.

## Discussion

ULs are a kind of disease with high incidence and unclear causes, which affects the normal life of women (McWilliams and Chennathukuzhi, 2017). Various genes may have been mutated in ULs via genome sequencing (Hodge et al., 2014; Mehine et al., 2014; Ajabnoor et al., 2018; Bray et al., 2019). Hence, there are more and more studies focused on SNPs of the genes in ULs, including polymorphisms of MED12, folk1, FANCA, genes for age at menarche (Yatsenko et al., 2017; Gulec Yilmaz et al., 2018; Ha et al., 2020; Ponomarenko et al., 2020). Many studies found that VitD deficiency might increase ULs risk (Davari Tanha et al., 2021; Islam et al., 2021; Vergara et al., 2021). Our previous study also showed that the levels of VitD in patients with ULs were lower than those in healthy controls (Li et al., 2020). It is speculated that there may be some mutations occurred in VitD anabolism-related genes and then influence the susceptibility to ULs. Therefore, this study investigated the relationship between polymorphisms of vitamin D anabolism related genes and ULs risks.

DHCR7 encodes 7-dehydrocholesterol reductase and the enzyme is involved in the conversion procedure of 7-dehydrocholesterol to cholesterol (Prabhu et al., 2016). 7-dehydrocholesterol is a precursor of VitD and can be changed into VitD3 under sunlight or ultraviolet radiation on the skin. NADSYN1 catalyzes the synthesis of NAD, a significant cofactor in multiple redox reactions, and may indirectly take part in the formation of VitD (Prabhu et al., 2016). The polymorphisms of DHCR7/NADSYN1 participate in susceptibility to many diseases, including deficiency of VitD, acute coronary syndrome, Alzheimer’s disease and ULs (Lu et al., 2012; Wise et al., 2014; Elbehairy et al., 2021; Liu et al., 2021). Three variants in DHCR7/NADSYN1 (rs11606033, rs3829251 and rs1790349) loci were tightly correlated to low levels of serum VitD (Lu et al., 2012; Elbehairy et al., 2021). Notably, it was reported that NADSYN1 rs2276360 G > C was related to serum 25(OH)D3 deficiency (Elbehairy et al., 2021). However, DHCR7 rs1044482 has not been found to be associated with VitD deficiency. At present, there are few studies on DHCR7/NADSYN1 polymorphisms in ULs. Liu et al. demonstrated that rs12800438 near DHCR7 was linked closely to the risk of ULs (Wise et al., 2014). For rs1044482, the allele frequency of T was 0.324 in the Asian population of NCBI’s dbSNP database, 0.350 in the control group and 0.431 in the ULs group in this study. For rs2276360, the allele frequency of C was 0.341 in the Asian population in the dbSNP database of NCBI, 0.321 in the control group and 0.372 in the ULs group in this study. DHCR7 rs1044482 was related to the occurrence of ULs and haploid analysis of DHCR7 rs1044482 and NADSYN1 rs2276360 showed that the combination of the two SNPs was significantly associated with susceptibility to ULs.

A study found that the incidence of ULs reached the peak in women aged 40–45 years (Yu et al., 2018). It was demonstrated that the incidence of ULs gradually increased with age (Pavone et al., 2018). Besides, aging is correlated with the reduction in intake, synthesis and function of VitD3 (Biesalski, 2021). In our study, although there was no difference in age between ULs group and control group, both of the two SNPs were related closely to susceptibility to ULs in older women (age ≥40 years). Therefore, age and site mutation may affect the level or function of vitamin D and thus induce the occurrence of hysteromyoma. In addition, many studies found that obesity is also a risk factor of ULs and women with higher BMI more tended to develop ULs (Giri et al., 2017; Qin et al., 2021). Compared with women with normal BMI, obese women produced more estrogen, making them prone to ULs (Cleary and Grossmann, 2009). However, DHCR7 rs1044482 was only statistically associated with the occurrence of ULs in women with normal BMI, not in women with high BMI in our study. The reason for the phenomenon might partly be the small number of women with high BMI. The proportion of participants in validation samples with >25.0 BMI was only 10.38%.

There are still some expectations in our study. In the future, this study will continue to recruit a large number of patients with ULs and normal women, and test the VitD level of each participant while analyzing the differences in DHCR7 rs1044482 and NADSYN1 rs2276360 between the two groups. Then, the source of participants will be enriched and more subjects will be recruited from multiple centers. Finally, potential mechanisms of DHCR7 rs1044482 increasing ULs risk should also be addressed in the following studies.

## Conclusion

In a word, the study suggested that DHCR7 rs1044482 C > T and NADSYN1 rs2276360 G > C might be related to the susceptibility to uterine leiomyomas in the Chinese population, especially in patients over 40 with 18.5–25.0 BMI.

## Data Availability

The original contributions presented in the study are publicly available. This data can be found here: Bioproject, accession number PRJNA807129; SRA database, accessions SRR18114348-SRR18114567.
