# Supplementary material for: Association of Vitamin D Anabolism-Related Gene Polymorphisms and Susceptibility to Uterine Leiomyomas
Source: Front Genet. 2022 Jun 20;13:844684. doi: 10.3389/fgene.2022.844684 (PMC9251306; doi:10.3389/fgene.2022.844684)
Supplement: Supplementary file 1 [file DataSheet1.doc]

**Supplementary table 1. TaqMan total reaction system and PCR conditions**

| Composition | Volume (μL) | Conditions |
| --- | --- | --- |
| TaqMan Genotyping Master Mix | 2 | 95℃, 10min; (95℃, 15s; 60℃, 1min) 40 cycles; 60℃ 30s; 4℃. |
| Probe (40X) | 0.04 |
| ddH_2_O | 1.96 |
| DNA | 1 |
| Total | 5 |

**Supplementary table 2. The sequencing results of 59 SNPs of vitamin D related metabolic genes in 110 ULs and 110 healthy controls**

| **SNP** | **Position** | **Alleles** | **Gene** | **Case (N=110)** | | |  | **Control (N=110)** | | | **HWE** | ***P*** |
| --- | --- | --- | --- | --- | --- | --- | --- | --- | --- | --- | --- | --- |
|  |  |  |  | 0/0 | 0/1 | 1/1 |  | 0/0 | 0/1 | 1/1 |  |
| NA | chr2: 219646963 | C > A | CYP27A1 | 110 | 0 | 0 |  | 109 | 1 | 0 | 0.962 | 1.000 |
| NA | chr2: 219647152 | C > T | CYP27A1 | 110 | 0 | 0 |  | 109 | 1 | 0 | 0.962 | 1.000 |
| rs748325824 | chr2: 219677025 | A > G | CYP27A1 | 109 | 1 | 0 |  | 110 | 0 | 0 | NA | 1.000 |
| rs181649030 | chr2: 219677076 | G > A | CYP27A1 | 107 | 3 | 0 |  | 107 | 3 | 0 | 0.885 | 1.000 |
| rs144018609 | chr2: 219677420 | G > A | CYP27A1 | 108 | 2 | 0 |  | 108 | 2 | 0 | 0.923 | 1.000 |
| rs121908099 | chr2: 219679132 | G > A | CYP27A1 | 110 | 0 | 0 |  | 109 | 1 | 0 | 0.962 | 1.000 |
| rs9016 | chr4: 72618296 | T > C | GC | 0 | 0 | 110 |  | 0 | 0 | 110 | NA | NA |
| rs4588 | chr4: 72618323 | G > T | GC | 56 | 50 | 4 |  | 52 | 46 | 12 | 0.704 | 0.116 |
| rs7041 | chr4: 72618334 | A > C | GC | 45 | 55 | 10 |  | 57 | 44 | 9 | 0.901 | 0.261 |
| rs760191055 | chr4: 72618365 | A > G | GC | 109 | 1 | 0 |  | 110 | 0 | 0 | NA | 1.000 |
| rs139133782 | chr4: 72620789 | G > A | GC | 109 | 1 | 0 |  | 110 | 0 | 0 | NA | 1.000 |
| rs4752 | chr4: 72622566 | A > G | GC | 100 | 10 | 0 |  | 96 | 13 | 1 | 0.463 | 0.479 |
| NA | chr4: 72634137 | A > G | GC | 110 | 0 | 0 |  | 110 | 0 | 0 | NA | NA |
| rs771550512 | chr4: 72634153 | G > T | GC | 109 | 1 | 0 |  | 110 | 0 | 0 | NA | 1.000 |
| rs180820099 | chr4: 72635115 | G > A | GC | 110 | 0 | 0 |  | 109 | 1 | 0 | 0.962 | 1.000 |
| NA | chr4: 72649747 | A > G | GC | 110 | 0 | 0 |  | 110 | 0 | 0 | NA | NA |
| rs3733359 | chr4: 72649774 | G > A | GC | 47 | 52 | 11 |  | 42 | 54 | 14 | 0.601 | 0.712 |
| rs34288833 | chr9: 137300003 | A > G | RXRA | 103 | 7 | 0 |  | 104 | 6 | 0 | 0.769 | 0.775 |
| rs201880753 | chr9: 137300095 | T > C | RXRA | 109 | 1 | 0 |  | 110 | 0 | 0 | NA | 1.000 |
| rs193137714 | chr9: 137300781 | C > T | RXRA | 108 | 2 | 0 |  | 109 | 1 | 0 | 0.962 | 0.562 |
| rs1805348 | chr9: 137328442 | G > A | RXRA | 109 | 1 | 0 |  | 110 | 0 | 0 | NA | 1.000 |
| rs202011621 | chr11: 14907321 | C > T | CYP2R1 | 110 | 0 | 0 |  | 109 | 1 | 0 | 0.962 | 1.000 |
| rs12794714 | chr11: 14913575 | G > A | CYP2R1 | 48 | 47 | 15 |  | 52 | 48 | 10 | 0.820 | 0.557 |
| NA | chr11: 14913633 | G > T | CYP2R1 | 109 | 1 | 0 |  | 110 | 0 | 0 | NA | 1.000 |
| NA | chr12: 48238390 | G > A | VDR | 110 | 0 | 0 |  | 110 | 0 | 0 | NA | NA |
| rs561663044 | chr12: 48238456 | G > T | VDR | 109 | 1 | 0 |  | 110 | 0 | 0 | NA | 1.000 |
| rs2229829 | chr12: 48238607 | G > T | VDR | 109 | 1 | 0 |  | 109 | 1 | 0 | 0.962 | 1.000 |
| rs201868713 | chr12: 48238682 | G > A | VDR | 110 | 0 | 0 |  | 109 | 1 | 0 | 0.962 | 1.000 |
| rs731236 | chr12: 48238757 | A > G | VDR | 107 | 3 | 0 |  | 102 | 8 | 0 | 0.692 | 0.215 |
| rs749838232 | chr12: 48238764 | G > A | VDR | 110 | 0 | 0 |  | 110 | 0 | 0 | NA | NA |
| NA | chr12: 48240137 | G > A | VDR | 110 | 0 | 0 |  | 109 | 1 | 0 | 0.962 | 1.000 |
| NA | chr12: 48272624 | T > G | VDR | 108 | 2 | 0 |  | 109 | 1 | 0 | 0.962 | 1.000 |
| rs527628548 | chr12: 48272631 | C > T | VDR | 110 | 0 | 0 |  | 109 | 1 | 0 | 0.962 | 1.000 |
| rs2228570 | chr12: 48272895 | A > G | VDR | 27 | 49 | 34 |  | 33 | 53 | 24 | 0.754 | 0.289 |
| NA | chr12: 58158156 | C > G | CYP27B1 | 110 | 0 | 0 |  | 109 | 1 | 0 | 0.962 | 1.000 |
| rs373672754 | chr12: 58158211 | A > T | CYP27B1 | 110 | 0 | 0 |  | 109 | 1 | 0 | 0.962 | 1.000 |
| rs190470241 | chr12: 58158820 | C > T | CYP27B1 | 109 | 1 | 0 |  | 110 | 0 | 0 | NA | 1.000 |
| rs368126466 | chr12: 58159128 | C > A | CYP27B1 | 110 | 0 | 0 |  | 109 | 1 | 0 | 0.962 | 1.000 |
| rs8176344 | chr12: 58159173 | C > G | CYP27B1 | 109 | 1 | 0 |  | 110 | 0 | 0 | NA | 1.000 |
| NA | chr12: 58159824 | G > A | CYP27B1 | 110 | 0 | 0 |  | 109 | 1 | 0 | 0.962 | 1.000 |
| NA | chr12: 58159836 | T > C | CYP27B1 | 110 | 0 | 0 |  | 109 | 1 | 0 | 0.962 | 1.000 |
| rs537715358 | chr12: 58160771 | C > T | CYP27B1 | 110 | 0 | 0 |  | 109 | 1 | 0 | 0.962 | 1.000 |
| rs703842 | chr12: 58162739 | A > G | METTL1 | 17 | 50 | 43 |  | 12 | 50 | 48 | 0.849 | 0.566 |
| rs762315043 | chr20: 32848301 | T > C | ASIP | 110 | 0 | 0 |  | 109 | 1 | 0 | 0.962 | 1.000 |
|  | chr20: 32856832 | G > T | ASIP | 109 | 1 | 0 |  | 110 | 0 | 0 | NA | 1.000 |
| rs772338707 | chr20: 32856839 | C > A | ASIP | 109 | 1 | 0 |  | 110 | 0 | 0 | NA | 1.000 |
| rs531438091 | chr20: 52773803 | G > A | CYP24A1 | 110 | 0 | 0 |  | 109 | 1 | 0 | 0.962 | 1.000 |
| rs2296239 | chr20: 52775528 | C > T | CYP24A1 | 14 | 53 | 43 |  | 17 | 49 | 44 | 0.586 | 0.897 |
| rs764570392 | chr20: 52779287 | A > G | CYP24A1 | 109 | 1 | 0 |  | 109 | 1 | 0 | 0.962 | 1.000 |
| rs76747058 | chr20: 52779338 | C > G | CYP24A1 | 105 | 4 | 1 |  | 106 | 4 | 0 | 0.846 | 0.605 |
| rs182993972 | chr20: 52782379 | T > C | CYP24A1 | 109 | 1 | 0 |  | 110 | 0 | 0 | NA | 1.000 |
| rs115260488 | chr20: 52786155 | C > T | CYP24A1 | 107 | 3 | 0 |  | 109 | 1 | 0 | 0.962 | 0.622 |
| rs2296241 | chr20: 52786219 | G > A | CYP24A1 | 39 | 49 | 22 |  | 31 | 58 | 21 | 0.507 | 0.429 |
| rs538419090 | chr20: 52788161 | T > C | CYP24A1 | 109 | 1 | 0 |  | 110 | 0 | 0 | NA | 1.000 |
| rs387907322 | chr20: 52788183 | C > G | CYP24A1 | 109 | 1 | 0 |  | 109 | 1 | 0 | 0.962 | 1.000 |
| NA | chr20: 52790100 | T > A | CYP24A1 | 110 | 0 | 0 |  | 109 | 1 | 0 | 0.962 | 1.000 |
| rs766316288 | chr2: 219647077 | GAG > | CYP27A1 | 109 | 1 | 0 |  | 110 | 0 | 0 | NA | 1.000 |
| rs757697462 | chr11: 71149931 | GACGTT > | DHCR7 | 109 | 1 | 0 |  | 110 | 0 | 0 | NA | 1.000 |
| NA | chr12: 58162657 | C > | METTL1 | 110 | 0 | 0 |  | 109 | 1 | 0 | 0.962 | 1.000 |

0/0 was homozygote without mutation; 0/1 was heterozygote with 1 mutation; 1/1 was homozygote with 2 mutations.

HWE, Hardy-Weinberg equilibrium; NA, not applicable.
